# A thermoreversible antibacterial zeolite-based nanoparticles loaded hydrogel promotes diabetic wound healing via detrimental factor neutralization and ROS scavenging

**DOI:** 10.1186/s12951-021-01151-5

**Published:** 2021-12-11

**Authors:** Yao Qi, Kun Qian, Jin Chen, Yifeng E, Yijie Shi, Hongdan Li, Liang Zhao

**Affiliations:** 1 School of Pharmacy, Jinzhou Medical University, Jinzhou, 121000 People’s Republic of China; 2 Department of Chemistry, Jinzhou Medical University, Jinzhou, 121000 People’s Republic of China; 3 Life Science Institute, Jinzhou Medical University, Jinzhou, 121000 People’s Republic of China

**Keywords:** Reactive oxygen species, Zeolite, Hydrogel, Pluronic F127, Chitosan

## Abstract

**Background:**

As recovery time of diabetic wound injury is prolonged by the production of detrimental factors, including reactive oxygen species (ROS) and inflammatory cytokines, attenuating the oxidative stress and inflammatory reactions in the microenvironment of the diabetic wound site would be significant.

**Experimental design:**

In our study, we prepared thermoreversible, antibacterial zeolite-based nanoparticles loaded hydrogel to promote diabetic wound healing via the neutralization of detrimental factors such as inflammatory cytokines and ROS.

**Results:**

The cerium (Ce)-doped biotype Linde type A (LTA) zeolite nanoparticles synergistically eliminated mitochondrial ROS and neutralized free inflammatory factors, thus remodeling the anti-inflammatory microenvironment of the wound and enhancing angiogenesis. Moreover, the thermoreversible hydrogel composed of Pluronic F127 and chitosan demonstrated strong haemostatic and bactericidal behavior.

**Conclusions:**

In conclusion, the obtained thermoreversible, antibacterial, zeolite-based nanoparticles loaded hydrogels represent a multi-targeted combination therapy for diabetic wound healing.

**Graphical Abstract:**

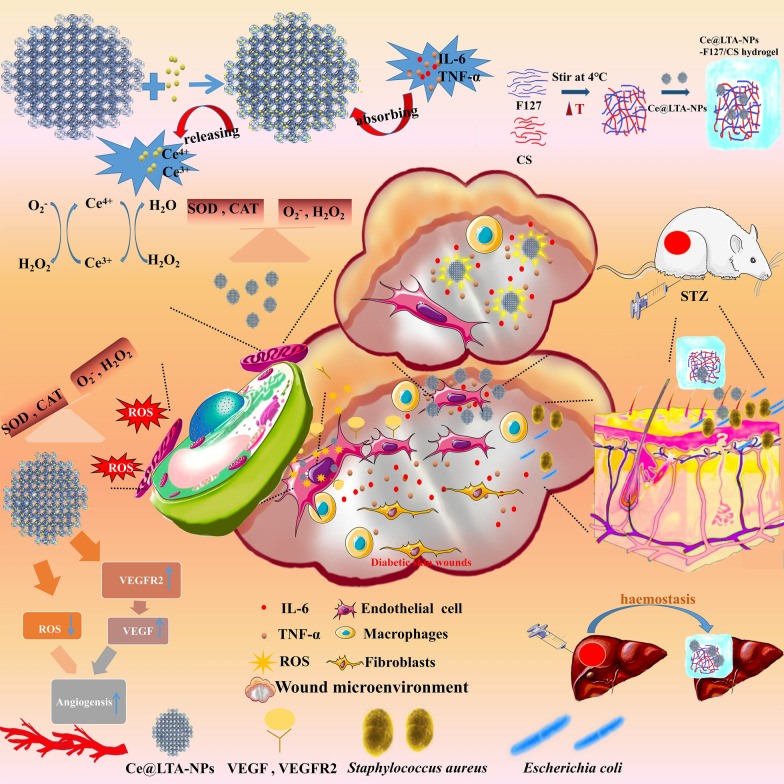

**Supplementary Information:**

The online version contains supplementary material available at 10.1186/s12951-021-01151-5.

## Introduction

Poor healing of chronic skin wounds caused by persistent hyperglycaemia is a serious complication of diabetes. According to reports, up to 15% of patients with diabetes [1] will eventually suffer from chronic skin wound ulceration, which is the most common cause of non-traumatic amputation worldwide. Chronic wounds caused by diabetes are exposed to the environment in the long-term and carry a high risk of infection [2]. Compared with the healing process of normal wounds [3], diabetic skin wounds clearly show obstacles in healing, slow and continuous inflammation, acute bacterial infections, the obstruction of new blood vessels and a lack of extracellular matrix (ECM) components. At present, there is no effective clinical method to treat diabetic wounds, and these wounds have become increasingly serious with high mortality and disability rates that threaten human health worldwide [4].

The key factors that affect diabetic wound healing are intricate [5]. Among them, the prolonged period of time spent in the pathological inflammatory microenvironment is hostile [6]. In chronic inflammatory wounds, most inflammatory cells are activated and substantially induce the secretion of pro-inflammatory cytokines (IL-6 and TNF-α) which are recruited to accumulate at the wound site. As a result, the large accumulation of inflammatory cytokines leads to serious insult [7, 8].

Oxidative stress is recognized as another key factor that leads to the delayed wound healing of diabetic wounds [9, 10]. The main manifestation of oxidative stress is an imbalance between reactive oxygen species (ROS) including superoxide anion, H_2_O_2_ and hydroxyl radicals, and endogenous antioxidants in the body [11]. A high concentration of glucose directly leads to the vast production of intracellular ROS secreted by the mitochondria in cells [12].This rapid generation of excessive mitochondrial ROS causes mitochondrial dysfunction, inhibited the binding of vascular endothelial growth factor (VEGF) and vascular endothelial growth factor receptor 2 (VEGFR2) [13, 14], leading to impaired angiogenesis, and subsequent endothelial cell chemotaxis and proliferation are restricted.

In addition to the large amounts of ROS and pro-inflammatory cytokines produced in diabetic wound sites [15], chronic bacterial infections also often occur, causing excessive local inflammation and extending the wound healing time. These wounds contain a variety of synergistic bacteria, such as *Staphylococcus aureus* and *Escherichia coli* [16]. Bacteria easily form biofilms at the skin injury site to resist the host immune response and antimicrobial treatment. In addition, it has been reported that *E.* *coli* infection may cause a reduction in the protein, oxyproline, collagen type I, and collagen type III contents in these wounds, consequently delaying their restoration.

**Fig. 1:**
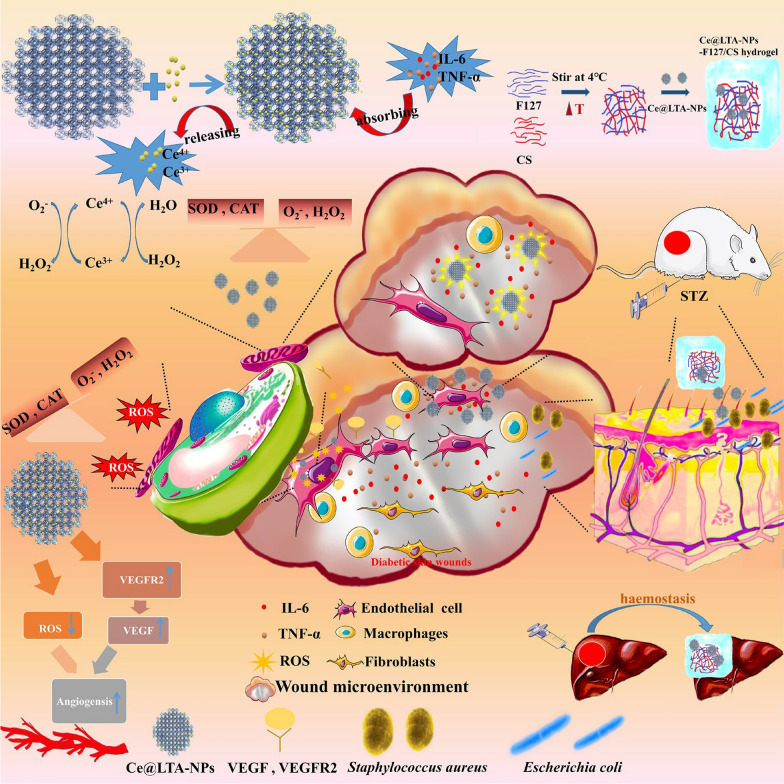
Ce@LTA-NPs loaded hydrogels composing of Pluronic F127 and CS (Ce@LTA-NPs-F127/CS hydrogel) were designed as a multi-target combination therapy for diabetic wound healing

In view of the potential effects of chronic inflammation, oxidative stress, and bacterial infections on the regulation of diabetic wound healing, we designed a thermoreversible hydrogel composed of Pluronic F127 and chitosan (CS) to encapsulate cerium (Ce) doped Linde type A (LTA) zeolite [17–19] based nanoparticles (Ce@LTA-NPs) as a multi-targeted combination therapy for treating diabetic wound healing [20, 21]. As shown in Fig. 1, the Ce@LTA-NPs loaded hydrogel composed of Pluronic F127 and CS (Ce@LTA-NPs-F127/CS hydrogel) showed a typical three-dimensional interlinked network structure, large porosity and local sustained release [22–24]. The Ce@LTA-NPs-F127/CS hydrogels exhibited thermoreversibility, strong antibacterial and haemostatic effects. Owing to their porous structure and large specific surface area, Ce@LTA-NPs had the ability to neutralize detrimental factors by adsorbing free inflammatory factors (TNF-α, IL-6) in the acute phase of wound healing, thus remodeling the wound microenvironment and preventing occurrence of a cytokine storm. Ce@LTA-NPs showed the ability to mimic the catalytic activities of superoxide dismutase (SOD) and catalase (CAT), thus eliminating free radicals including ROS induced by hyperglycaemia and regulating the oxidative state in the microenvironment of the wound site. Finally, Ce@LTA-NPs-F127/CS hydrogel enhanced the migration and proliferation of endothelial cells by regulating VEGF, VEGFR2 and PI3K, and accelerated diabetic wound healing by neutralizing harmful factors and scavenging ROS.

### Materials and animals

Ce(SO_4_)_2_·(H_2_O)_4_ (98.0%) was acquired from Shanghai Yien Chemical Technology Co., Ltd. (China), Na_2_SiO_3_ was acquired from Wuxi Yatai United Chemical Industry, NaAlO_2_ was obtained from Liaoning Quanrui Reagent Co., Ltd. (China), NaOH (98.0%) was acquired from Tianjin Fengfan Chemical Reagent Co., Ltd. (China), chitosan (CS, deacetylation degree 50%) was obtained from Macklin Biological Product Co., Ltd. (China); and Pluronic F127 was obtained from Yuan Ye Biotechnology Co., Ltd. (China). All other chemicals were of reagent grade and used as received. Antibodies against VEGF, VEGFR2, MMP2, and PI3K (1:500) were purchased from WanLeiBio (Shenyang, China). IL-6 and TNF-α were purchased from Abcam (ab179570, ab183218) and the goat anti-rabbit IgG/HRP secondary antibody was obtained from EarthOx Life Sciences (Millbrae, CA, USA), S. aureus (ATCC 25923) and Escherichia coli (ATCC 25922) were obtained from the American Type Culture Collection (ATCC).

### Cell culture

Human umbilical vein endothelial cells (HUVECs) were obtained from the Cell Line Bank of the Chinese Academy of Sciences (Shanghai, People’s Republic of China). The murine macrophage RAW264.7 cell line was purchased from the cell bank of the Chinese Type Culture Collection of Wuhan University. Cells were cultured in DMEM/F12 medium containing 10% fetal bovine serum, and the cell culture conditions were as follows: 95% humidity; with 5% CO_2_; in a 37 °C incubator. To mimic the diabetic environment in vitro, high glucose stimulated HUVECs (HG-HUVECs) were often employed and obtained by treating HUVECs for 72 h with high glucose (HG; 30 mmol/L), changing the media every 24 h, as previously described.

### Animals

Male Sprague–Dawley (SD) rats (220–250 g) were provided by Jinzhou Medical University. The experimental protocol was performed with the approval of the Institutional Animal Care and Use Committee of Jinzhou Medical University and followed the National Guidelines for Animal Protection.

### Synthesis and characterization of Ce@LTA-NPs-F127/CS hydrogel

NaOH (15.0 g) and NaAlO_2_ (12.0 g) were added to 90 mL of water. Then, 12.0 g of Na_2_SiO_3_ was dissolved in 50 mL of water and added dropwise to the above solution. After stirring for 30 min, 0.3 g of CeSO_4_ was added to the reaction system. After another 30 min of stirring, the gel was transferred to a steel autoclave reactor with Teflon inner vessel and heated at 100 °C for the required time. After heating, the product was separated from the whole system by centrifugation at 15,000 rpm for 30 min. Then the product was washed with water through a dialysis bag until the desired conductivity was reached. The product was also synthesized in the similar route without the addition of Ce for the contrastive study.

To obtain the Ce@LTA-NPs-F127/CS hydrogels, CS and Ce@LTA-NPs were dispersed in 20% W/V F127, and then continuously stirred overnight at 4 °C. TEM images were photographed on Tecnai G2 F20 Field Emission Transmission Electron Microscope from America FEI Company, at a voltage of 200 kV to observe the morphology of the product. Powder X-ray diffraction (XRD) Spectrograms were collected to identify the phase. The X-ray Photoelectron Spectroscopy (XPS) analysis was performed on an ESCALAB 250Xi instrument from Thermo Fisher Scientific, USA. The chemical bond structure was examined using FTIR spectroscopy (Shimadzu, Japan) and UV–vis spectroscopy (Shimadzu, Japan). The particle size and surface charge were measured by dynamic light scattering (Zetasizer Nano ZS; Malvern Instruments, Malvern, UK). BET data were acquired on a Tristar II 3020 surface area analyser (Micromeritics Instrument Corporation, USA) with nitrogen adsorption–desorption isotherms. Ce@LTA-NPs were weighed accurately in an ceramic hermetic sample pans and sealed with a lid, Then measurements of the samples were performed on differential scanning calorimeter (DSC) (DSC-60, Shimadzu, Inc. Japan) with a heating rate of 10 °C/min from 25 to 600 °C under a nitrogen atmosphere with a flow rate of 120 °C/min.

The rheological properties of the F127 hydrogels, F127/CS hydrogels and Ce@LTA-NPs-F127/CS hydrogels were assessed using a rotational rheometer (Anton Paar MCR92). Briefly, the storage modulus (G′) and loss modulus (Gʺ) of the hydrogels were measured under different conditions, including a change from 0 to 50 °C. The viscosities of hydrogels were also measured at different temperatures (0–50 °C) and shear rates (0–100 1/s, 37 °C).

### Safety evaluation of Ce@LTA-NPs in vivo and in vitro

Cell viability of HUVECs treated with Ce@LTA-NPs was assessed via MTT assay, according to our previous reports. Ce@LTA-NPs were co-incubated with HUVECs, MTT solution was added and dimethyl sulfoxide (DMSO) was used to dissolve the formed formazan. Finally, the absorbance was measured at 490 nm. In terms of haemolysis analysis, 3 mL whole blood from SD rats was treated with 3.2% sodium citrate as an anticoagulant, and collected by centrifugation at 2500 rpm for 10 min, the upper plasma was then discarded. The resulting lower layer of red blood cells was washed 3 times with 2 mL of physiological saline until the washing solution was colorless. Next, 1 mL of red blood cells was mixed with 49 mL of normal saline to obtain a 2% (v/v) red blood cell suspension. Sample no. 6 was normal saline as a negative control group, and sample no. 7 was pure water and used as a positive control group. Sample numbers 1–5 were 2.5 mL red blood cell suspension samples containing different concentrations of Ce@LTA-NPs (0.2, 0.4, 1, 2, and 3 mg/mL, respectively) in 15 mL centrifuge tubes in a 37 °C water bath for incubation for 3 h. Finally, after centrifugation at 1500 rpm for 15 min, the supernatant was collected and its absorbance was measured at 570 nm. To evaluate the in vivo safety of Ce@LTA-NPs, H&E staining [25] was performed using heart, liver, lung, spleen and kidney tissue sections after i.v. administration of 1 mL of saline, or 1 mL of various amounts of Ce@LTA-NPs (6 or 12 mg/kg per day) for 15 days.

### ROS elimination analysis

A cell reactive oxygen detection kit was used to explore whether the cerium ion had ROS scavenging effects due to its change in valence state. HUVECs were stimulated with 30 mmol/L glucose for 72 h or 600 μmol/L H_2_O_2_ for 4 h, and 10 μg/mL or 50 μg/mL Ce@LTA-NPs were added and incubated with HUVECs pretreated with high glucose or H_2_O_2_ for 24 h. The oxidant-sensing fluorescent probe 2,7-dichlorofluorescein diacetate (DCFH-DA; Sigma-Aldrich, St Louis, MO, USA) was used to analyze the intracellular ROS. SOD and CAT assay kits were used to measure the superoxide anion and hydrogen peroxide scavenging activities of Ce@LTA-NPs by measuring the absorbance at 560 nm and 240 nm.

### Free inflammatory factor neutralization analysis

To prove that Ce@LTA-NPs had the ability to adsorb substances, a BET experiment was carried out in vitro. The pore size, surface area and adsorption capacity of Ce@LTA-NPs were detected. Immunofluorescence experiments verified the co-localization of TNF-α and IL-6 with Ce@LTA-NPs in macrophages stimulated by 100 ng/mL lipopolysaccharide (LPS). Western blot experiments detected the expression of inflammatory factors in culture medium of LPS stimulated macrophages. The culture medium was collected followed by the addition of 50 μg/mL Ce@LTA-NPs for continous incubation at 4 °C overnight. Finally, the supernatant was collected by centrifugation at 120,000*g*, the concentrations of proteins were determined by BCA assay and the inflammatory factors (IL-6, TNF-α) were determined by Western blotting. Finally, an immunofluorescence experiment was used to analyze the expression of inflammatory factors in skin tissue sections from diabetic rats.

### Angiogenesis analysis

To evaluate the angiogenesis level of HG-HUVECs treated with Ce@LTA-NPs, CCK-8 assays, scratch-wound, transwell assays and tube formation assays were performed in vitro. For the scratch-wound assay, HG-HUVECs were seeded in a 6-well cell culture plate, and the cell monolayer was scraped in a straight line with a p200 pipette. After 50 μL of Ce@LTA-NPs (50 μg/mL) was incubated with the cells, the HG-HUVECs migrated towards the centre of the well, and their migration was evaluated by measuring the gap distance. For the transwell assay, Ce@LTA-NPs were placed into a transwell insert precoated with basement membrane Matrigel and incubated with HG-HUVECs for 12 h in an incubator at 37 °C, with 5% CO_2_. Migration was determined by counting cells on the lower surface to determine the cell migration rate. In addition, tube formation of HG-HUVECs was investigated according to previous reports [26], Ce@LTA-NPs were incubated with HG-HUVECs in wells preloaded with Matrigel for 8 h, and the tube length was measured and the branching points were counted [27].

### Antibacterial and haemostatic activity of Ce@LTA-NPs-F127/CS hydrogels

To evaluate the antibacterial activities of the F127/CS hydrogels and Ce@LTA-NPs-F127/CS hydrogels, 100 μL of F127/CS hydrogel and Ce@LTA-NPs-F127/CS hydrogel were incubated with bacterial suspensions containing 1 × 10^6^ CFU of either *E. coli* or *S. aureus* to observe the bacterial growth in LB-agar growth plates [28]. The optical density (OD) of each bacterial suspension was measured with a microplate reader at 600 nm. We also evaluated the haemostatic properties of hydrogels using a rat model of massive hepatic haemorrhage by determining the amount of bleeding after applying the hydrogels in the bleeding site of liver and comparing the results with the untreated wounds.

### Diabetic rat wound model evaluation

Type 1 Diabetes Mellitus was induced in SD rats, and wounds with a diameter of 2 cm were established as a diabetic wound model in a sterile environment. F127 hydrogels, F127/CS hydrogels and Ce@LTA-NPs-F127/CS hydrogels were separately placed on the surface of the wounds twice daily for 2 weeks and the wound areas were statistically analysed on the third, seventh, and fourteenth days. Wound sections were subjected to haematoxylin and eosin (H&E) [29] and immunohistochemistry analysis to evaluate the healing abilities of the wounds by observing inflammatory cell infiltration and blood vessel formation. Masson’s trichrome staining was used to observe the morphological changes and collagen content in the wound tissues.

### Statistical analysis

All data are presented as means standard deviation (SD). Comparison between groups was performed by one-way ANOVA analysis. *p < 0.05, **p < 0.01, and ***p < 0.001 were considered to be statistically significant.

## Results

### Characterization of the Ce@LTA-NPs-F127/CS hydrogels

**Fig. 2:**
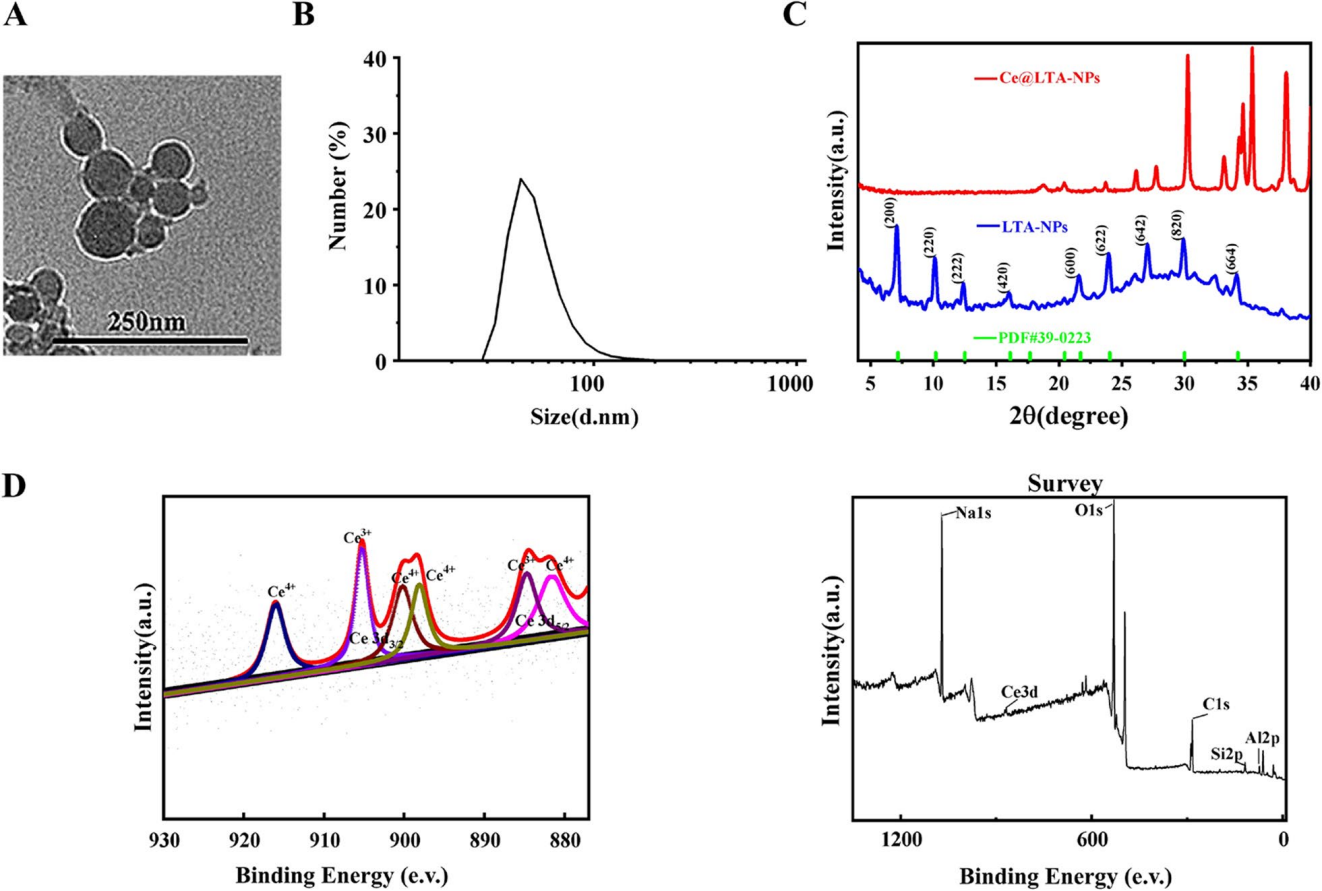
Morphology and structure characterization of Ce@LTA-NPs. **A** TEM images of Ce@LTA-NPs. **B** Particle size distribution of Ce@LTA-NPs measured by DLS. **C** The XRD of Ce@LTA-NPs. **D** The XPS spectrum for the elements Ce, Al, Si, and Na in Ce@ LTA-NPs

TEM image verified the morphology of Ce@LTA-NPs [30]. As shown in Fig. 2A, it showed that Ce@LTA-NPs were uniformly dispersed in nano-sized scale. The Malvern particle size analyzer was used to further measure the particle size distribution of Ce@LTA-NPs. As shown in Fig. 2B, the particle size distribution of Ce@LTA-NPs was in the range of 33–164 nm. To further study the crystallization of the Ce@LTA-NPs, we performed XRD characterization by comparing the spectrum of the product with that of the LTA zeolite material [31, 32]. The XRD pattern results (Fig. 2C) showed that the XRD pattern of the prepared Ce@LTA-NPs matched well with the XRD pattern of the cubic phase LTA zeolite (JCPDS file number 39-0223) with diffraction peak position. However, the diffraction peaks of the first four crystal plane could not be observed after the addition of Ce, which revealed relationship between the main pore channel and the settled Ce ions. At the same time, the crystallinity of the high angle peak is enhanced in the XRD pattern of the prepared Ce@LTA-NPs which showed the doped Ce improved the crystallization degree of secondary structure units. To fully identify the structure of the Ce@LTA-NPs, XPS, FTIR, and UV methods were used to determine their composition. The XPS (Fig. 2D) charts proved the elemental surface presence of Na, Si, and Al in the carrier, and Ce was also found in the final composition. High-resolution XPS spectroscopy verified the existence of two mixed Ce valence states, Ce^3+^ and Ce^4+^. The peaks at 881.6, 898.1, 900.2 and 916.1 eV were associated with Ce^4+^, while the peaks at 884.6 and 903.2 eV were associated with Ce^3+^ [33, 34].This proved that the Ce^4+^/Ce^3+^-co-doped LTA-NPs was successfully prepared, and Ce accounted for 7.56% of the total amount of Ce@LTA-NPs.

The zeta potential analysis confirmed that Ce@LTA-NPs was -31.4 ± 2.5 mV, satisfying colloidal steady-state conditions. As shown in Additional file 1: Fig. S1A and B, the FTIR spectrum and UV–visible absorption spectrum of Ce@LTA-NPs also showed both Ce species such as Ce^3+^ and Ce^4+^, which was consistent with the previous reports [35]. FTIR spectrum further confirmed that the absorption peak at approximately 531 cm^−1^ for the vibration peak of the Ce–O bond was clearly observed, and the broad peak at approximately 3436 cm^−1^ corresponded to the O–H on the surface of the Ce@LTA-NPs was also visible. The absorption peaks at approximately 1001 cm^−1^ and 738 cm^−1^ represented the anti-symmetric symmetric stretching vibration of Si–O, and the anti-symmetric stretching vibration of Si–O–Si in the zeolite. Due to the charge transfer transitions O^2−^–Ce^3+^ and O^2−^–Ce^4+^, the coexistence of both Ce^3+^ and Ce^4+^ species exhibited specific broad and overlapping absorption bands between approximately 200 and 300 nm in the UV–visible absorption spectrum [36]. To further investigate the thermal stability of Ce@LTA-NPs, a DSC experiment (Additional file 1: Fig. S1C) was carried out. The results showed that when the temperature of the DSC curve of Ce@LTA-NPs was in the range of 100–200 °C, the DSC curve showed that the water in different coordination states disappeared. When the temperature was in the range of 200–600 °C, there were no endothermic or exothermic peaks. These thermal peaks proved the thermal stability of Ce@LTA-NPs.

### Ce@LTA-NPs biocompatibility evaluation

**Fig. 3:**
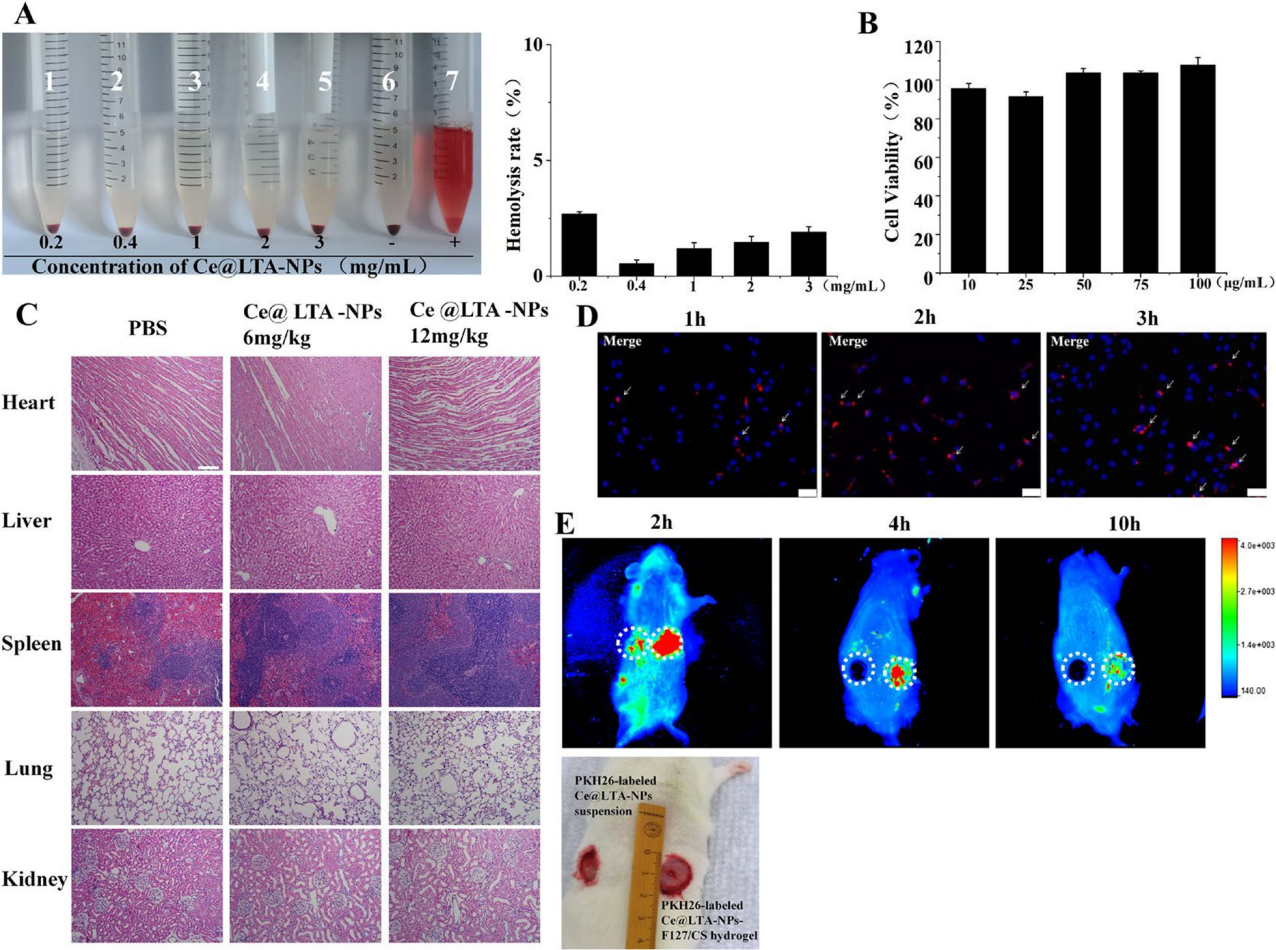
Biocompatibility evaluation of Ce@LTA-NPs **A** Haemolysis determined by observing whether the red blood cells were ruptured after treatment with different concentrations of Ce@LTA-NPs. **B** The viability of HUVEC cells at 24 h after treatment with different concentrations of Ce@LTA-NPs. **C** H&E staining of heart, liver, lung, spleen and kidney tissue sections after i.v. administering a single dose of 1 mL of saline and 1 mL of various amount of Ce@LTA-NPs (6 mg/kg and 12 mg/kg per day) for 15 days. The scale bar is 100 μm. **D** Representative fluorescence images of PKH26-labelled Ce@LTA-NPs incubated with HG-HUVECs for 1 h, 2 h and 3 h. The scale bar is 50 μm. **E** Representative fluorescence images (overlaid with photograph) of diabetic wound site which receiving the treatment of PKH26-labelled Ce@LTA-NPs suspension and PKH26-labelled Ce@LTA-NPs-F127/CS hydrogel at 2 h, 4 h, and 10 h. The round circle indicated the diabetic wound site. All Data were expressed as mean ± SD (n = 3)

To evaluate the possibility of using Ce@LTA-NPs as therapeutic agents, they must possess good biological compatibility in vivo and in vitro. The results of haemolysis test (Fig. 3A) showed that the Ce@LTA-NPs had good blood compatibility. All of the erythrocytes in the positive control group treated with pure water were broken up. In contrast, when the concentration of the Ce@LTA-NPs reached 3 mg/mL, the haemolysis rate of the red blood cells treated with the Ce@LTA-NPs was less than 5% and still maintained a normal cell structure and morphology, indicating that the Ce@LTA-NPs were a safe nanomaterial [37, 38]. The MTT results (Fig. 3B) showed that when the concentration of Ce@LTA-NPs reached 100 μg/mL, the Ce@LTA-NPs did not exhibit obvious cytotoxicity, and the cell survival rate remained above 85%. The H&E staining results (Fig. 3C) showed that after rats were exposed to the Ce@LTA-NPs for 15 days, no toxicity or side effects on the major organs were observed. Compared with the sham group (saline treatment), Ce@LTA-NPs at 12 mg/kg did not induce obvious histological differences in the major organs, suggesting that no significant toxicity was induced by the Ce@LTA-NPs.

### Cellular uptake in vitro and local distribution in diabetic wound sites

According to the previous reports [39, 40], the uptake of Ce@LTA-NPs in HG-HUVECs and the prolonged release of Ce@LTA-NPs in diabetic wound sites were investigated in vivo and in vitro. When 50 μg/mL Ce@LTA-NPs were labeled with the dye PKH26 and then incubated with HG-HUVECs for 1 h, 2 h or 3 h, as shown in Fig. 3D, the red fluorescence emitted by PKH26-labeled Ce@LTA-NPs were gradually increased over time, indicating that increasing amounts of Ce@LTA-NPs were taken up by cells and can be effectively delivered into the appropriate compartment, confirming the feasibility of the Ce@LTA-NPs to regulate the biological functions of HG-HUVECs. To prove the local targeting capacity and sustained retention of the Ce@LTA-NPs-F127/CS hydrogels in the diabetic wound site, the suspension containing PKH26-labelled Ce@LTA-NPs were injected subcutaneously around the wounds of SD rats, and PKH26-labelled Ce@LTA-NPs-F127/CS hydrogels were also covered in the wound site. As shown in Fig. 3E, it was found that after 2 h of administration of PKH26-labelled Ce@LTA-NPs suspension, the red fluorescence representing the presence of Ce@LTA-NPs was almost invisible in the diabetic wound site, indicating that the majority of Ce@LTA-NPs was removed from the wound site and showed fast clearance. In contrast, with the extension of time, PKH26-labelled Ce@LTA-NPs were slowly released from the hydrogel and accumulated in the wound site for a longer time. Therefore, red fluorescence emitted by PKH26-labelled Ce@LTA-NPs in hydrogels was gradually enhanced with the extension of time. The strong red fluorescence emitted by PKH26-labelled Ce@LTA-NPs-F127/CS hydrogels was obviously observable after 4 h and still visible at 10 h, indicating that the Ce@LTA-NPs-F127/CS hydrogels prolonged the retention of Ce@LTA-NPs in the wound site, thus avoiding frequent, repeated administration and promoting long-term therapeutic effects.

### Ce@LTA-NPs inhibited the production of ROS by restoring the integrity of the mitochondria and mimicking catalytic activities in vivo

**Fig. 4:**
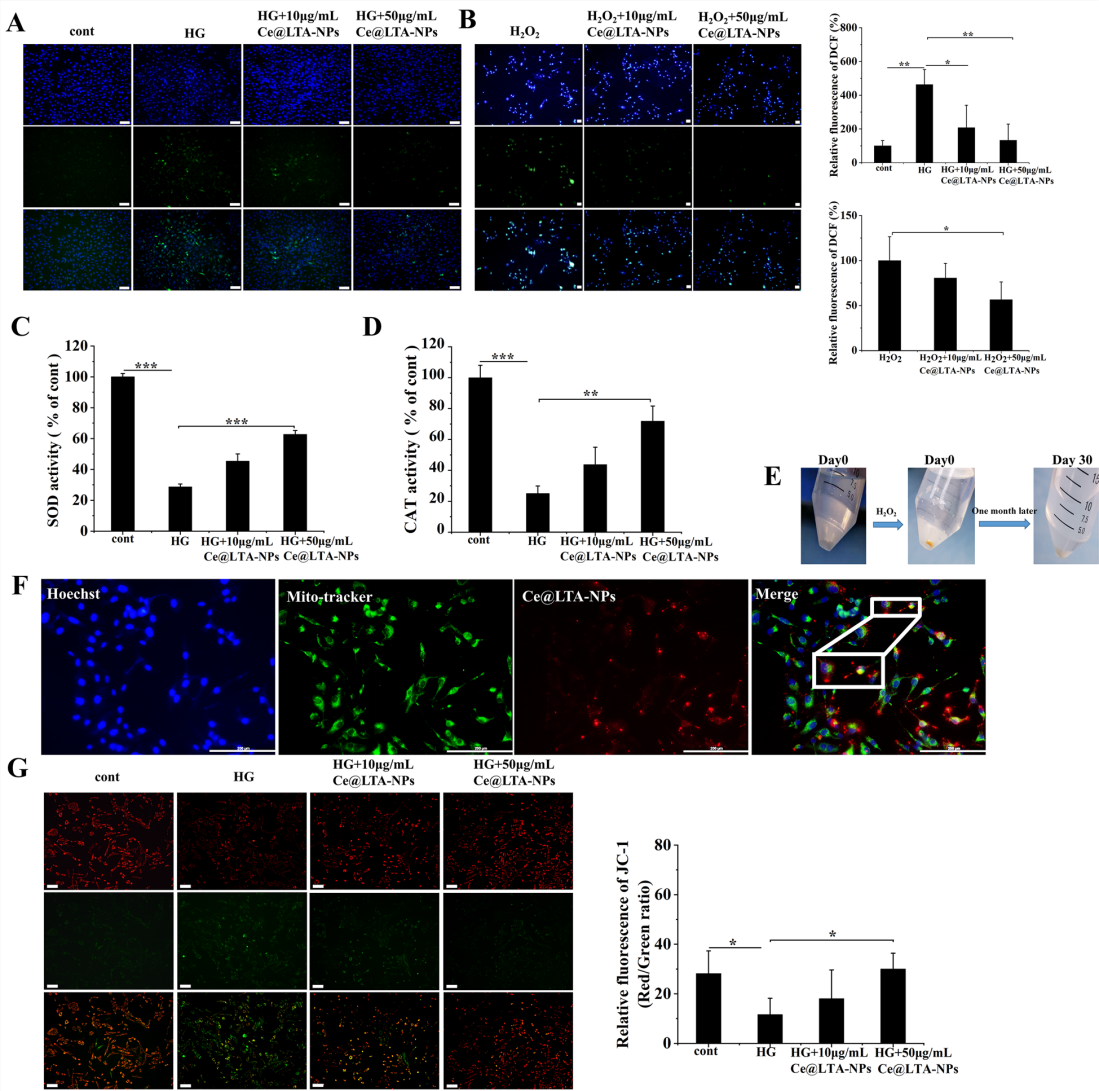
ROS inhibition of Ce@LTA-NPs via targeted mitochondria and enhanced catalytic activities. Untreated normal HUVECs were set as control group. **A** ROS generation in HG-HUVECs cells treated with different concentrations of Ce@LTA-NPs. Data are expressed as mean ± SD (n = 3), *p < 0.05, **p < 0.01. The scale bar is 100 μm. Relative fluorescence ratio (%) of DCF was determined by calculating the ratio of fluorescence intensity of DCF in group to fluorescence intensity of DCF in normal HUVECs (control group). **B** ROS generation after HUVECs stimulated by H_2_O_2_. The data were expressed as the mean ± SD (n = 3), *p < 0.05, and the scale bar is 50 μm. Relative fluorescence ratio (%) of DCF was determined by calculating the ratio of fluorescence intensity of DCF in group to fluorescence intensity of DCF in H_2_O_2_ treated group. **C** The relative SOD activity. The data were expressed as the mean ± SD (n = 3), ***p < 0.001. Relative SOD activity (%) was determined by calculating the ratio of level of SOD in group to the level of SOD in normal HUVECs (control group). **D** The relative CAT activity. The data were expressed as the mean ± SD (n = 3), **p < 0.01, ***p < 0.001. Relative CAT activity (%) was determined by calculating the ratio of level of CAT in group to the level of CAT in normal HUVECs (control group). **E** Determination of the autocatalytic activity of Ce@LTA-NPs. **F** The colocation of mitochondria and Ce@LTA-NPs in cells. The scale bar is 200 μm. **G** The change of mitochondrial membrane potential of HG-HUVECs cells treated with Ce@LTA-NPs, the scale bar is 100 μm, and the data are expressed as mean ± SD (n = 3), *p < 0.05

To explore whether Ce had a certain ROS scavenging effect due to changes in the valence state [41], DCFH-DA was used to detect intracellular ROS levels [42]. Figure 4A showed that when HUVECs were stimulated with 30 mmol/L glucose for 72 h, the concentration of ROS was higher in HG-HUVECs than that in normal HUVECs, indicating that HG induced the generation of ROS. Ce@LTA-NPs significantly decreased the ROS levels in a dose-dependent manner, as determined by quantification of the fluorescence intensity of DCF. Moreover, it was also found in Fig. 4B that the Ce@LTA-NPs inhibited the H_2_O_2_-mediated generation of ROS in HUVECs. Next, we examined the SOD and CAT mimetic catalytic activities of Ce@LTA-NPs [43]. It demonstrated that in Fig. 4C and D high concentrations of Ce@LTA-NPs exhibited the enhanced SOD and CAT catalytic activities and catalyzed the decomposition of reactive oxygen species such as the superoxide radicals and hydrogen peroxide. As shown in Fig. 4E, the autocatalytic activity of Ce@LTA-NPs was evaluated by treating Ce@LTA-NPs with 1 M H_2_O_2_ solution [44]. When H_2_O_2_ solution was added, the color of obtained powder of Ce@LTA-NPs after high-speed centrifugation changed from colorless to yellow, indicating that more Ce^4+^ species were produced. As time progressed, when H_2_O_2_ was exhausted, the color of Ce@LTA-NPs changed from yellow to white, which proved that more Ce^3+^ was produced.

It had been reported that the induction of ROS was related to the integrity of the mitochondria [45] and internalization of some NPs could specifically occur at the mitochondria to regulate ROS generation [46].We therefore explored the co-localization between Ce@LTA-NPs and the mitochondria to evaluate their effects on mitochondrial integrity and function. As shown in Fig. 4F, the red fluorescence of the PKH26-labelled Ce@LTA-NPs and the green fluorescence of the mitochondrial indicator were co-localized as yellow dots [47]. Mitochondrial membrane potential analysis (Fig. 4G) using the dye JC-1 demonstrated that compared with the normal HUVECs (control group), the HG-HUVEC group showed weaker red fluorescence and stronger green fluorescence in the mitochondria. Furthermore, the red/green ratio was significantly decreased. This result indicated that HG induced a loss of mitochondrial membrane potential and triggered mitochondrial damage. In contrast, after treatment with Ce@LTA-NPs, the membrane potential was restored to a higher level; thus, enhanced red fluorescence and lower green fluorescence was observed. These results proved that the Ce@LTA-NPs can target the mitochondria, improving mitochondrial function by increasing the mitochondrial membrane potential.

### Ce@LTA-NPs neutralized detrimental factors via absorption in vitro and in vivo

**Fig. 5:**
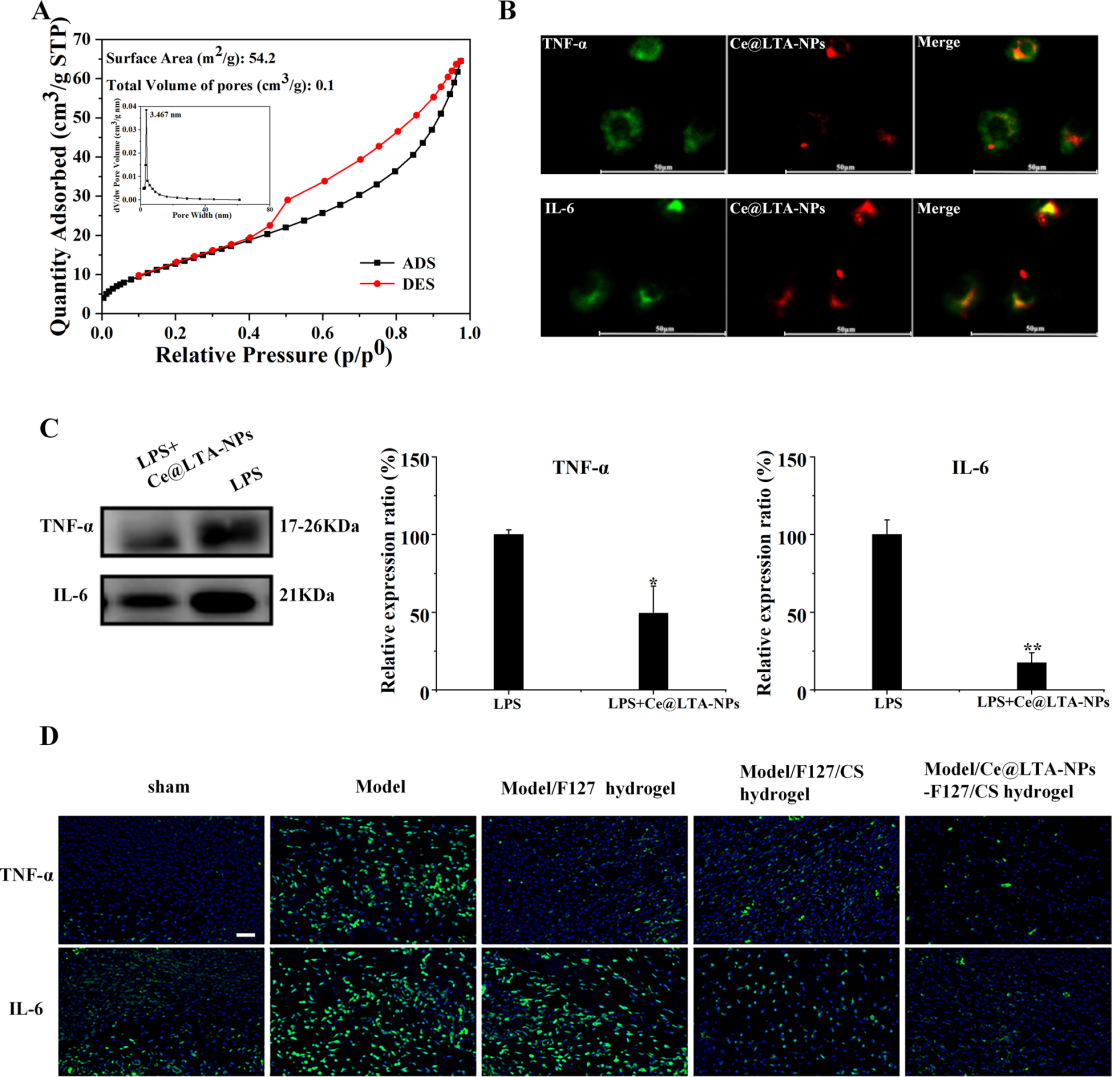
Ce@LTA-NPs neutralized inflammatory factors in vivo and in vitro. **A** BET analysis for Ce@LTA-NPs. **B** Co-localization analysis of inflammatory factors and Ce@LTA-NPs in macrophages exposed to LPS. The scale bar is 50 μm. **C** Western blot analysis of the expression levels of TNF-α and IL-6 in culture medium from LPS stimulated macrophages treated with 50 μg/mL Ce@LTA-NPs. The data are expressed as the mean ± SD (n = 3), *p < 0.05, **p < 0.01, Relative expression ratio (%) of TNF-α and IL-6 was determined by calculating the expression level of TNF-α and IL-6 in group to the expression level of TNF-α and IL-6 in LPS treated group. **D** Immunofluorescence assay of the expression of TNF-α and IL-6 in diabetic wound treated with F127 hydrogel, F127/CS hydrogel and Ce@LTA-NPs-F127/CS hydrogel. The scale bar is 50 μm

As the adsorption ability of material is highly related to their surface area, pore volume, and porous structure [48, 49], we first investigated the nitrogen adsorption/desorption isotherms and pore sizes of the Ce@LTA-NPs using BET surface area analysis. The results in Fig. 5A showed that the Ce@LTA-NPs demonstrated a proper surface area with a typical IV isotherm. The N_2_ adsorption of Ce@LTA-NPs gave the BET surface area of 54.2 m^2^/g, and the pore volume of 0.1 cm^3^/g. The results showed that Ce@LTA-NPs exhibited good absorption capacity as nanomaterials. In Fig. 5B showed that when PKH26-labelled Ce@LTA-NPs were incubated with LPS stimulated macrophages, the red fluorescence of the PKH26-labelled Ce@LTA-NPs was co-localized with the green fluorescence emitted by inflammatory factors, indicating that Ce@LTA-NPs had the ability to adsorb free inflammatory factors and neutralize the explosive release of harmful factors due to their adsorption capacity. In Fig. 5C, it showed the ability of Ce@LTA-NPs to adsorb inflammatory factors (IL-6 and TNF-α) by Western blotting. Macrophages (5 × 10^5^ cells/mL) were cultured in a 6-well plate and stimulated with 100 ng/mL LPS for 24 h. Next, the cell culture medium was centrifuged at 2000*g* for 10 min, 10,000*g* for 30 min at 4 °C to remove cells and debris, Then 50 μg/mL Ce@LTA-NPs were added for continuous incubation at 4 °C for 12 h. Finally, after removing the free Ce@LTA-NPs by centrifugation at 120,000*g* for 120 min at 4 °C in a Type CS150GX ultracentrifuge (Hitachi, Koki, Co., Ltd, Japan), the supernatant was collected, and the inflammatory factors (IL-6, TNF-α) were determined by western blotting. It was found that the protein expression levels of the inflammatory cytokines IL-6 and TNF-α in the culture medium of Ce@LTA-NPs treated group were significantly decreased compared with those in the medium of the untreated LPS stimulated macrophages. Finally, we also confirmed that Ce@LTA-NPs-F127/CS hydrogel decreased expression levels of the inflammatory cytokines IL-6 and TNF-α in diabetic wound tissue, which attributed to the ability of Ce@LTA-NPs to adsorb inflammatory factors (IL-6 and TNF-α) (Fig. 5D).

### Effects of Ce@LTA-NPs on angiogenesis

**Fig. 6:**
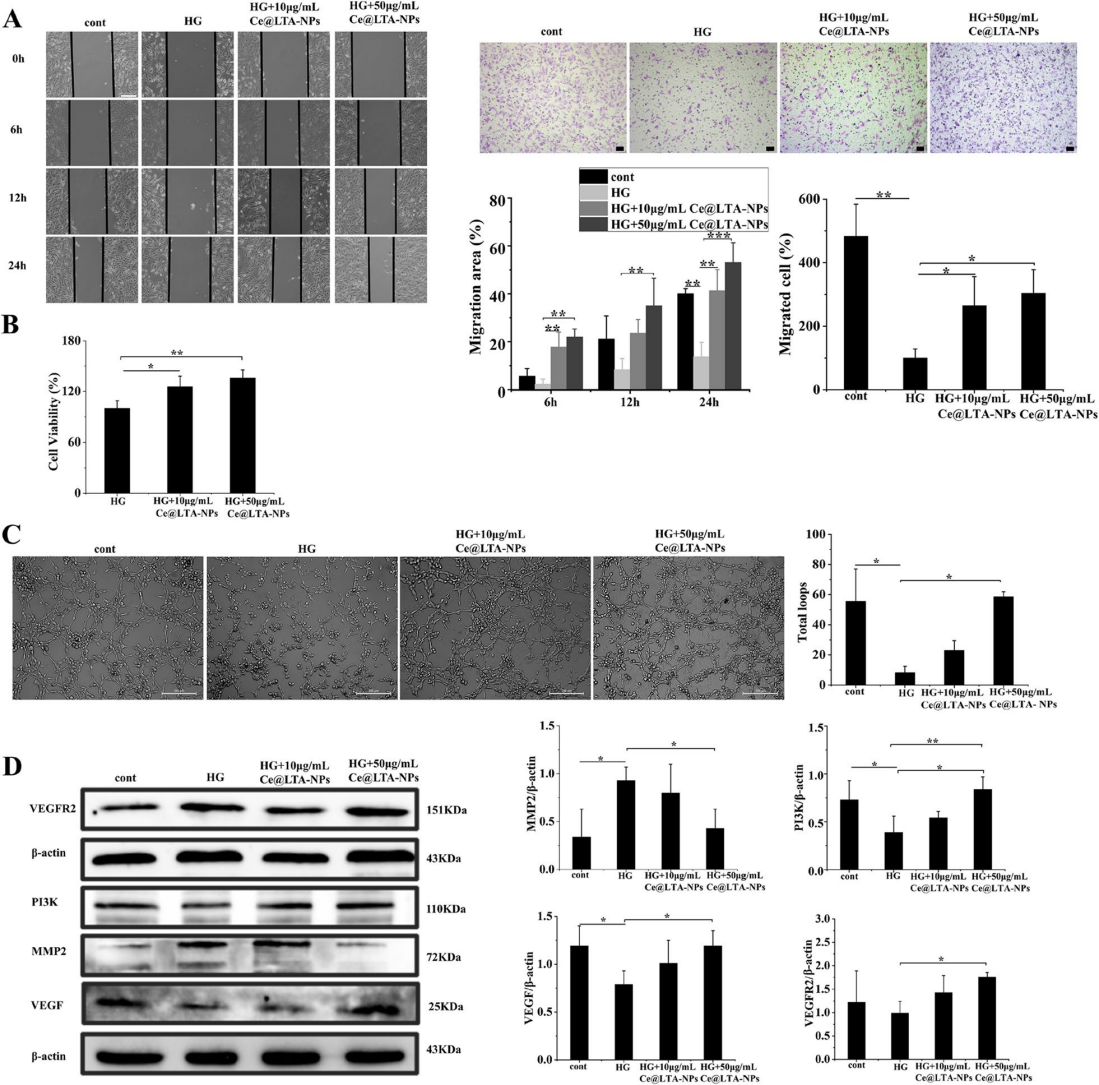
In vitro angiogenesis test. Normal HUVECs were set as control group. **A** Cell scratch assay in HG-HUVECs treated with different concentrations of Ce@LTA-NPs for 6, 12, and 24 h (scale bar: 200 μm). Transwell cell migration assay in HG-HUVECs treated with different concentrations of Ce@LTA-NPs for 12 h (scale bar: 50 μm). These data are represented as the means ± SD (n = 3) *p < 0.05, **p < 0.01, ***p < 0.001. The ratio of migrated cells (%) was determined by calculating the ratio of the number of migrated cells in group to the number of migrated cells in untreated HG-HUVECs group. **B** CCK-8 results of HG-HUVECs cells treated with different concentrations of Ce@LTA-NPs for 24 h; these data are represented as the means ± SD (n = 3) *p < 0.05, **p < 0.01. **C** In vitro tube formation assay in HG-HUVECs treated with different concentrations of Ce@LTA-NPs for 8 h (Scale bar: 200 μm); data are represented as the means ± SD (n = 3) *p < 0.05. **D** Western blot analysis of the expression levels of VEGFR2, VEGF, PI3K, MMP2 in HG-HUVEC treated with 10 μg/mL, 50 μg/mL Ce@LTA-NPs for 24 h. These data are represented as the means ± SD (n = 3) *p < 0.05, **p < 0.01

Figure 6A indicated that the cell migration ability of HG-HUVECs was lower than that of normal HUVECs. Treatment with 10 μg/mL or 50 μg/mL Ce@LTA-NPs accelerated the migration ability of HG-HUVECs. In particular, the wound healing rate in Ce@LTA-NPs (50 μg/mL) treated HG-HUVECs after 12 h was approximately 4.2 times higher than that of the untreated HG-HUVEC, and at 24 h, it was more than 3.8 times that of the untreated HG-HUVEC group. In the Transwell assay, Ce@LTA-NPs enhanced the migration of HG-HUVEC, as assessed by quantifying the number of migrating cells in a dose-dependent manner. To explore Ce@LTA-NPs induced proliferation in HG-HUVECs [50], HG-HUVECs were treated with different concentrations of Ce@LTA-NPs, which significantly promoted cell proliferation, showing 1.35-fold enhanced proliferation rates within 24 h compared with that in the untreated HG-HUVEC group set at 100% (Fig. 6B). In addition, as shown in Fig. 6C, after incubation with the cells for 8 h, the capillary-like loop that formed in the HG-HUVECs group was significantly reduced compared to that in the normal HUVECs group. In contrast, the number of loops and vascular branches in the Ce@LTA-NPs-treated HG-HUVECs group was significantly increased compared with those in the untreated HG-HUVECs group. Thus, it was fully proven that the Ce@LTA-NPs had the ability to promote angiogenesis in vitro. The Western blot results (Fig. 6D) revealed that the expression levels of VEGF, VEGFR2 and PI3K in the Ce@LTA-NPs-treated group were significantly higher than those in the untreated HG-HUVECs group. In addition, HG induced overexpression of the MMP2 protein, thus causing excessive degradation of extracellular matrix and delaying wound healing. The expression of the MMP2 protein in this group was significantly reduced given the high concentration of Ce@LTA-NPs.

### Optimization of the thermoreversible antibacterial hydrogels

**Fig. 7:**
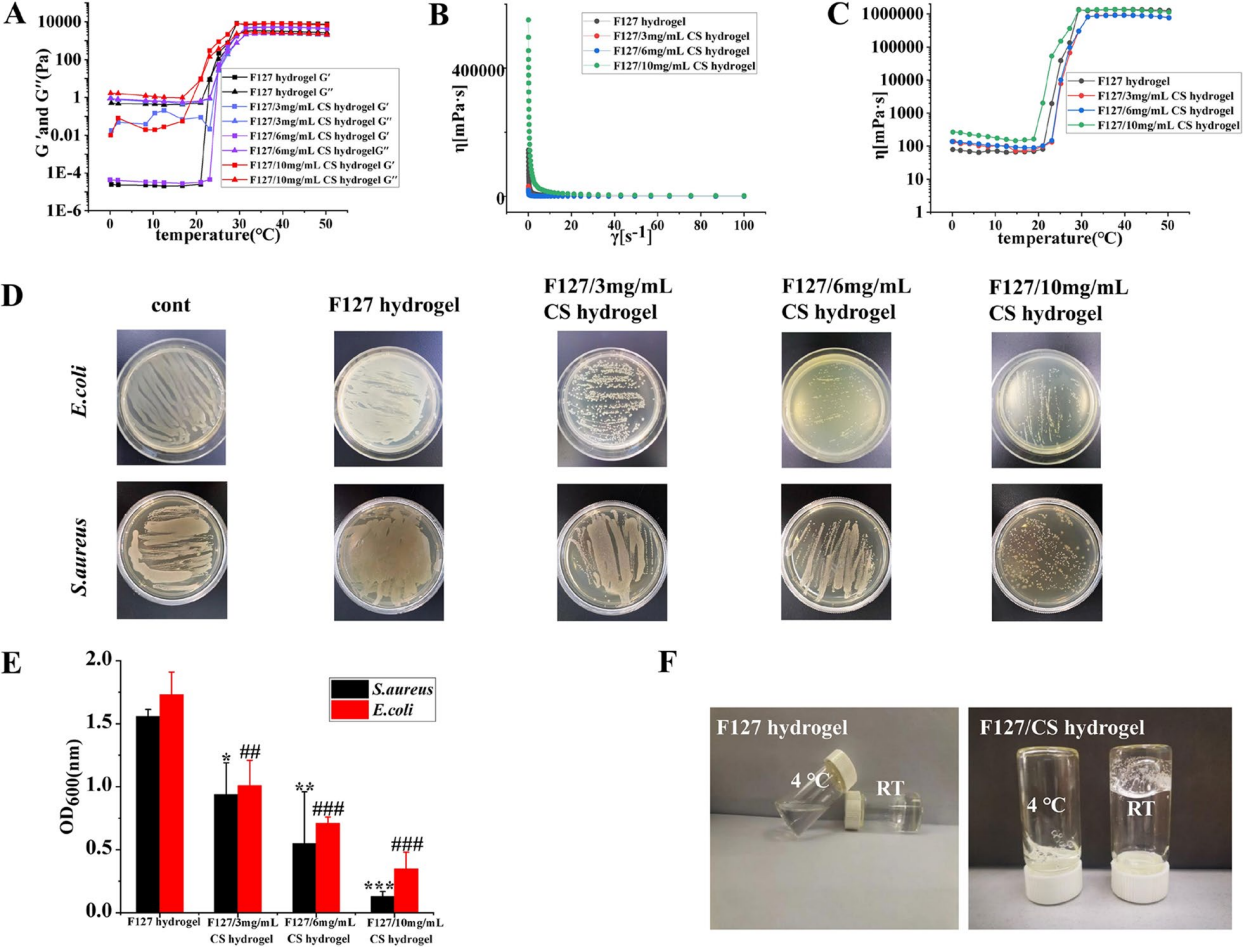
Rheological and antibacterial properties of thermally responsive F127/CS hydrogels. **A** Storage and loss modulus in F127/CS hydrogels with different ratio of F127 and CS with changes of temperature. **B** The viscosity of F127/CS hydrogels containing different ratio of F127 and CS with changes of shear stress. **C** The viscosity of F127/CS hydrogels containing different ratio of F127 and CS with changes of temperature. **D** Photographs of surviving bacteria clones of *E. coli* and *S. aureus* co-cultured with F127/CS hydrogels containing different ratio of F127 and CS on agar plates. untreated *E. coli* and *S. aureus* were set as control group. **E** Determination of the viability of the bacterial suspension containing F127/CS hydrogels containing different ratio of F127 and CS. These data are represented as the means ± SD (n = 3). ^##^p < 0.01, ^###^p < 0.001 versus OD_600_ nm of *E. coli* treated with F127 hydrogel, *p < 0.05, **p < 0.01, ***p < 0.001 versus OD_600_ nm of *S. aureus* treated with F127 hydrogel. **F** Hydrogels state at room temperature and 4 °C

**Fig. 8:**
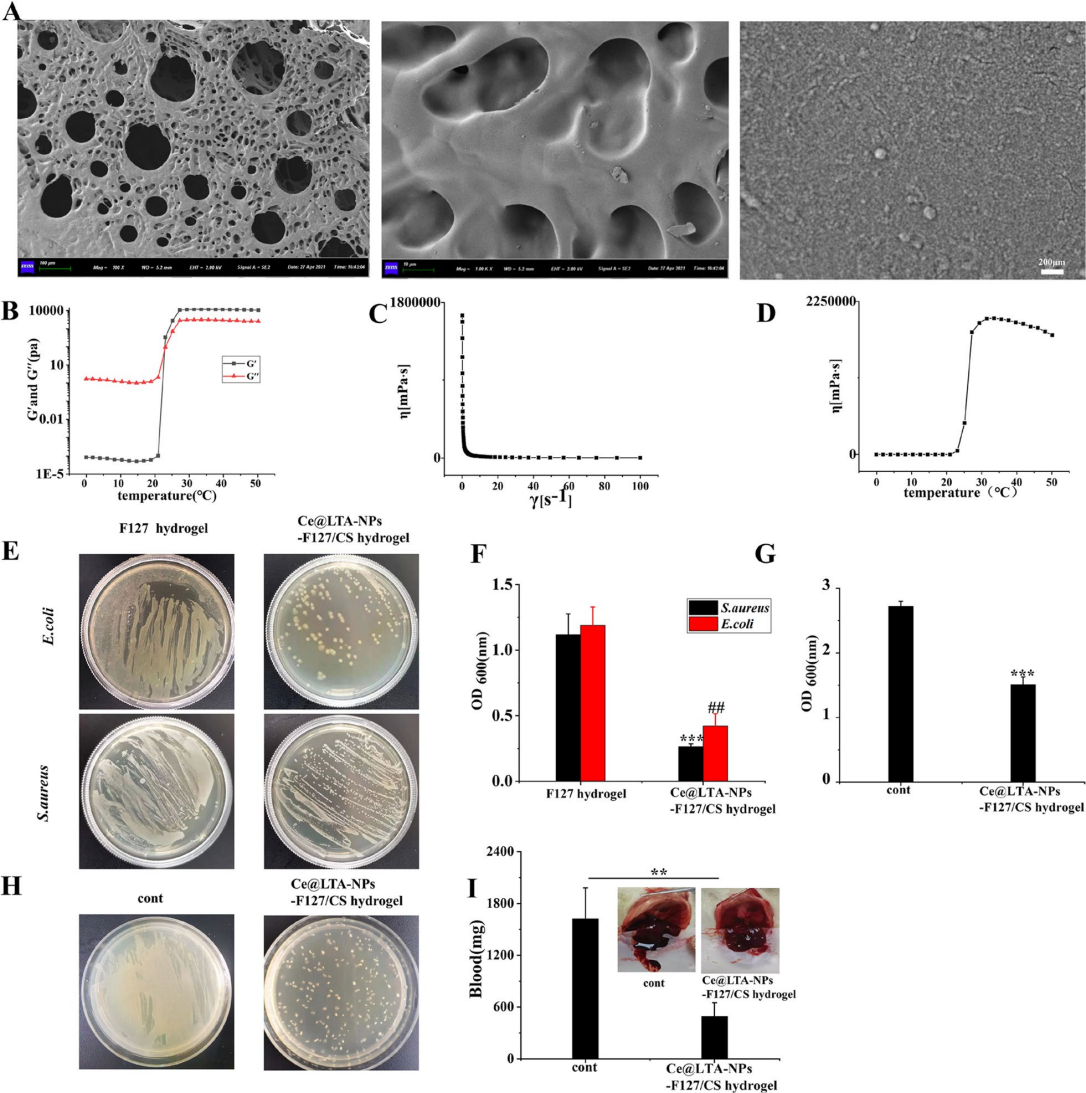
Morphology, rheology and antibacterial properties of Ce@LTA-NPs-F127/CS hydrogels. **A** Representative SEM image of the Ce@LTA-NPs-F127/CS hydrogels. **B** Storage and loss modulus of Ce@LTA-NPs-F127/CS hydrogels with change of temperature. **C** The viscosity of Ce@LTA-NPs-F127/CS hydrogels with the changes of shear stress. **D** The viscosity of Ce@LTA-NPs-F127/CS hydrogels with the changes of temperature. **E** Photos of *E. coli* and *S. aureus* culture plates after exposure to Ce@LTA-NPs-F127/CS hydrogels. **F** OD_600nm_ of bacterial suspension treated with Ce@LTA-NPs-F127/CS hydrogels. These data represent three separate experiments and are presented as the mean values ± SD. ^##^p < 0.01, ***p < 0.001. **G** OD_600nm_ of bacteria in wound exudates from diabetic wound tissues treated with Ce@LTA-NPs-F127/CS hydrogels. OD_600_ nm of bacteria in wound exudates from untreated diabetic wound tissues was set as control group. These data represent three separate experiments and are presented as the mean values ± SD, ***p < 0.001. **H** Photos of bacteria in wound exudates from diabetic wound tissues treated with Ce@LTA-NPs-F127/CS hydrogels. Photo of bacteria in wound exudates from untreated diabetic wound tissues was set as control group. **I** Haemorrhagic analysis in liver treated with Ce@LTA-NPs-F127/CS hydrogels. The amount of bleeding after applying the hydrogels in the bleeding site of liver and comparing the results with the untreated bleeding site (control group). These data represent three separate experiments and are presented as the mean values ± SD, **p < 0.01

To optimize the thermoreversible antibacterial hydrogels, the viscoelastic and rheological properties of the F127/CS hydrogels were investigated by measuring its mechanical responses [51]. Figure 7A represented the thermal scanning rheological observations of both the elastic and viscous modulus of F127/CS hydrogels after storage at 4 °C. The obtained gel containing different concentrations of CS showed high Gʺ values at low temperatures as compared with G′ values, but upon heating, the elastic and viscous moduli increased, and crossover of G′ and Gʺ occurred, indicating the transition from a solution to an elastic network. Figure 7B showed the flow curves that were determined at variable shear rates and temperatures. The viscosities of the gels decreased rapidly with slight increases in the shear rate. As the shear rate continued to increase gradually, the viscosity of the gels decreased slowly, exhibiting shear thinning behavior. In Fig. 7C, the viscosity of the gel gradually increased with increasing temperature. When the gel reached a temperature close to the temperature of human skin, the viscosity of the hydrogels did not increase but instead showed a pronounced plateau. As shown in Fig. 7D and E, to find the F127/CS composite hydrogel with the best antibacterial effects, different concentrations of CS were selected to investigate the antibacterial activity against gram-negative bacteria and gram-positive bacteria. It demonstrated that the F127/CS hydrogels significantly inhibited the growth of *E. coli* and *S. aureus* with increasing concentrations of CS. The best bactericidal concentration of CS loaded in the hydrogels was 10 mg/mL. Therefore, based on the rheological and bacteriostasis results, 10 mg/mL CS was finally mixed into F127 (20% w/v) to obtain the F127/CS hydrogels. To obtain Ce@LTA-NPs-F127/CS hydrogels, CS and Ce@LTA-NPs were dispersed in 15 mL of distilled water followed by the quick addition of 3 g of F127 under continuous stirring at 4 °C. SEM analysis of Ce@LTA-NPs-F127/CS hydrogels (Fig. 8A) revealed a three-dimensional network shape with a uniform and porous morphology, and Ce@LTA-NPs was loaded at the surface of hydrogels. In Fig. 8B–D, the addition of Ce@LTA-NPs did not affect the microstructure of the gel system, and the Ce@LTA-NPs-F127/CS hydrogels showed rheological behaviour that was similar to that of the F127/CS hydrogel. As shown in Fig. 8E and F, the antibacterial properties of hydrogels were tested according to the method reported in the literature [52]. The Ce@LTA-NPs-F127/CS hydrogel exhibited remarkable antibacterial activity with very low OD_600_ values of 0.42 and 0.263 against *E. coli* and *S. aureus*, respectively [53]. To evaluate the antibacterial activity of Ce@LTA-NPs-F127/CS hydrogels in vivo, the bacteria from the wound site after application of the hydrogel were cultured, and the optical density (OD) of the bacterial suspension was detected at 600 nm [54]. As shown in Fig. 8G and H, compared with the significant bacterial proliferation observed in the untreated diabetic wounds, Ce@LTA-NPs-F127/CS hydrogels covered the wound and exerted enhanced antibacterial effects at lower OD. We next evaluated haemostatic properties of Ce@LTA-NPs-F127/CS hydrogels using a haemorrhagic liver SD rats model. It was found that Ce@LTA-NPs-F127/CS hydrogels showed excellent hemostatic properties, and the total blood loss in the hydrogel group was much lower than that in control group (493.5 ± 157.3 mg vs 1623.1 ± 359.1 mg) (Fig. 8I).

### Ce@LTA-NPs-F127/CS hydrogels accelerated diabetic wound healing in type 1 diabetic rats

**Fig. 9:**
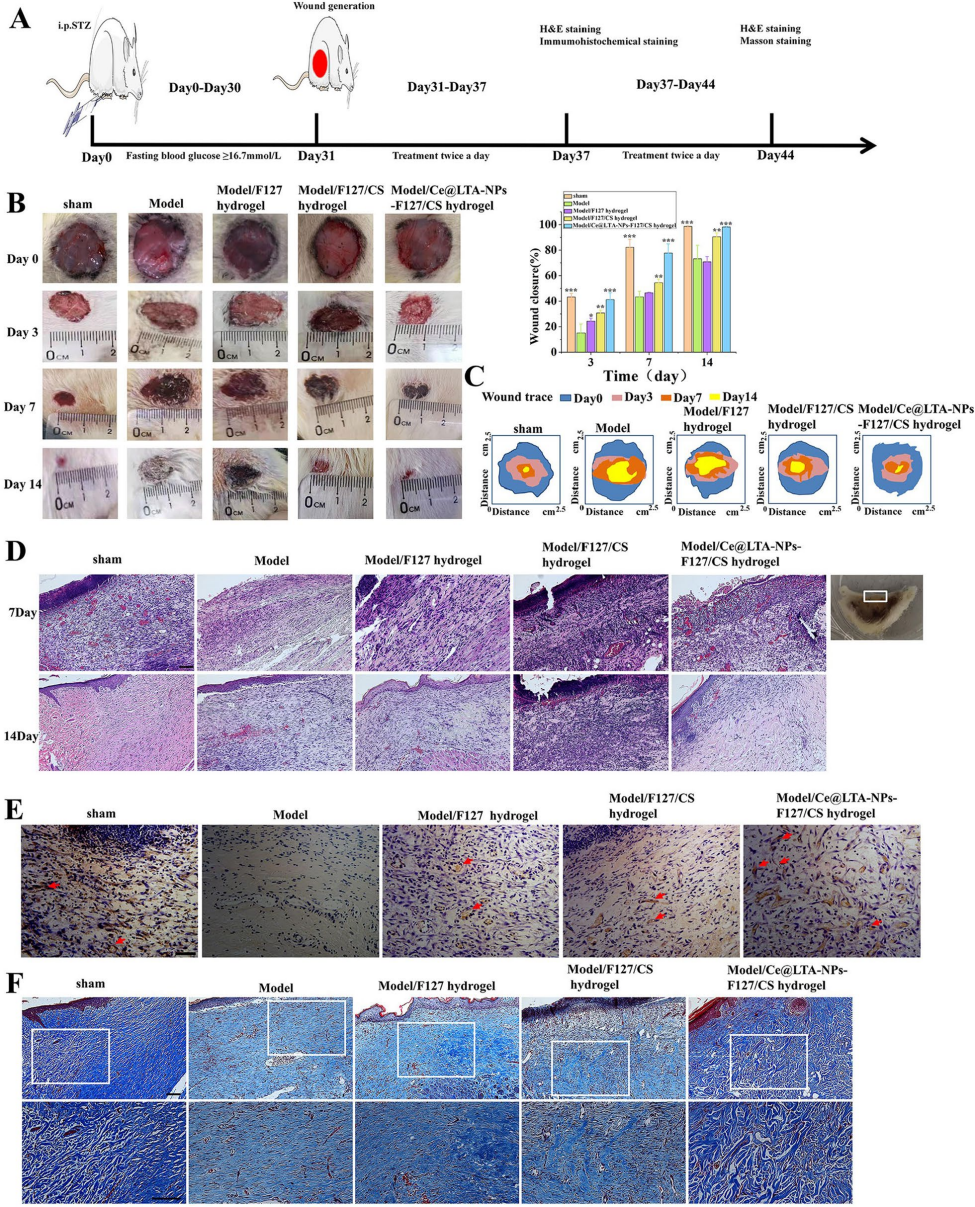
Healing process and histological evaluation of diabetic wound promoted by Ce@LTA-NPs-F127/CS hydrogels dressing. The untreated wound in the normal rat was set as sham group. The untreated wound in a type 1 diabetic rat was set as model group. **A** Design chart of animal experiment. **B** Representative images and wound healing rate of full-thickness skin defect in a type 1 diabetic rat model treated with F127 hydrogels, F127/CS hydrogels, Ce@LTA-NPs-F127/CS hydrogels after operation. These data are represented as the means ± SD (n = 3). *p < 0.05, **p < 0.01, ***p < 0.001. **C** Traces of the wound closure during 14 days for each treatment. **D** Representative H&E staining images of wound sections treated with F127 hydrogels, F127/CS hydrogels, Ce@LTA-NPs-F127/CS hydrogels at 7th day and 14th day after operation. The scale bar is 100 μm. **E** Immunohistochemistry assay of CD31 expression in diabetic wound sites treated with F127 hydrogels, F127/CS hydrogels, Ce@LTA-NPs-F127/CS hydrogels at 7th day. Vascular endothelial cell (CD31) and cell nucleus were stained brown and blue. Neovascularization was identified by positive CD31 staining (brown). The scale bar is 50 μm. **F** Masson’s trichrome staining of wound sections treated with F127 hydrogels, F127/CS hydrogels, Ce@LTA-NPs-F127/CS hydrogels at 14th day after operation. The scale bar is 100 μm

As shown in Fig. 9A, the potential role of the Ce@LTA-NPs-F127/CS hydrogels in the regulation of the diabetic wound process was further explored using type 1 diabetic SD rats by examining the wound closure rate and wound healing quality. The results in Fig. 9B and C showed that the untreated diabetic wound site as the model group was not completely closed, epithelialization was not complete, and the epithelium was thinner within 14 days. Ce@LTA-NPs-F127/CS hydrogels improved the wound closure rate by demonstrating complete closure. The effects of the Ce@LTA-NPs-F127/CS hydrogels on the pathological structure of rat skin wounds were investigated using H&E staining. The results of Fig. 9D showed that more neutrophil infiltration and loose granulation tissue on the wound edge with the formation of fewer fibroblasts were observed in the model group. In contrast, after Ce@LTA-NPs-F127/CS hydrogel treatment, there were only a few inflammatory cells at the wound site, and more granulation tissue and blood vessels were formed. As shown in Additional file 2: Fig. S2, we measured the wound length of each group after 7 days and 14 days. It was clear that the Ce@LTA-NPs-F127/CS hydrogel group had the shortest wound length on the 7th and 14th day as compared with model group, F127 hydrogel and F127/CS hydrogel treated groups. Subsequently, we further evaluated the angiogenesis and collagen synthesis in the wound through CD31 immunohistochemistry assay and Masson staining assay [55–57]. In Fig. 9E, it was found that the number of new blood vessels in the Ce@LTA-NPs-F127/CS hydrogels treated group was significantly higher than that in the model group on the 7th day. Similarly, In Fig. 9F, the Masson's trichrome staining observations showed more blue collagen fibres formation and their neat arrangement on the 14th day in the Ce@LTA-NPs-F127/CS hydrogel treated group as compared to the model group and the pure hydrogel groups. All results indicated that Ce@LTA-NPs-F127/CS hydrogels accelerated wound healing and improved the quality of diabetic wound healing by inhibiting inflammation, enhancing angiogenesis and regulating collagen formation.

## Discussion

In summary, diabetic wounds are usually locked in an inflammatory state that prevents proliferation. This is a complex process involving coordination between multiple cell types, such as neutrophils and macrophages [58]. When inflammation occurs, these cells are recruited to the wound to play a coordinated anti-inflammatory role. However, diabetes can cause hyperglycaemia-related metabolic disorders and directly damage the functions of the skin and inflammatory cells, leading to a slow and continuous inflammatory response [59]. Thus, understanding and controlling the initiation and resolution of inflammation is essential for the treatment of pathological wound healing. Traditional anti-inflammatory treatment strategies mainly include inhibiting the expression of inflammatory cytokines/enzymes and regulating the transition of macrophages from an inflammatory phenotype to a repair phenotype [60]. However, attack by inflammatory factors on the wound will never stop. As the efficacy of drugs that inhibit the expression of inflammatory factors decreases, inflammatory factors cause continuous damage to diabetic wounds. Therefore, although most medical treatments have good results during the early stage, they fail to improve wound healing in the later stage. Thus, there is an urgent need to develop a treatment strategy to alleviate the aggravation of wounds that is caused by the continuous and harmful erosion of high-level harmful factors and compensate for the lack in drug treatment. For this reason, we focused on the inflammatory factors that are produced in the wound site and, proposed a “nano-adsorption” strategy to neutralize free inflammatory factors in the wound site.

The metal nanomaterials currently reported to be applied to wounds are prepared by chemical synthesis. However, they face major obstacles such as health hazards and material instability [61]. Zeolites are characterized by a large specific surface area, hard framework, modifiable internal and external surface end groups, and good biocompatibility [62]. The crystal size of zeolite is generally on the order of micrometres, and its growth has undergone a process from silicon tetrahedrons to the formation of secondary structural units, quasicrystals, and finally micron-order crystals. Therefore, the application of zeolite as a nano-sized agent in organisms is limited. To overcome this limitation, we used an in situ synthesis method to intercept the zeolite before the formation of micron-scale crystals to prepare the nano-scaled Ce@LTA-NPs. We proved that Ce@LTA-NPs had important application prospects in the medical field due to their good biological safety and their ability to adsorb harmful substances [63]. Because of their regular and uniform pore structure and unique three-dimensional crystal structure, Ce@LTA-NPs demonstrated a unique adsorption capacity, the ability to adsorb explosive inflammatory factors induced by acute inflammation in diabetic wounds, acceleration of the transition from inflammation to proliferation, and remodeling of the wound microenvironment. On the other hand, due to Ce^3+^/Ce^4+^ doping on the surface of Ce@LTA-NPs, it exhibited the mimic activities of superoxide dismutase and catalase in cells to eliminate the excessive generation of ROS. Furthermore, Ce@LTA-NPs could target mitochondria and reverse mitochondrial dysfunctions caused by hyperglycaemia. Ce@LTA-NPs as nano-biomaterials can be loaded into the biocompatible, antibacterial temperature-sensitive F127/CS hydrogels [64, 65] and applied to the diabetic wound surface. Ce@LTA-NPs significantly improved the proliferation, migration and angiogenesis of HG-HUVECs [66]. Furthermore, in vivo studies confirmed that the Ce@LTA-NPs-F127/CS hydrogels promoted the formation of granulation tissue, re-epithelialization and collagen remodeling at the wound site, thereby accelerating the healing process of diabetic wounds.

All of these findings indicate that Ce@LTA-NPs-F127/CS hydrogels will provide an effective clinical method for the comprehensive repair of diabetic wound injury. After hydrogel formation, Ce@LTA-NPs-F127/CS hydrogels was converted to elastic and adhesive sheets in order to use in preclinical and clinical applications. Hydrogels we prepared can maintain the morphological and mechanical stability necessary for wound dressing. The better adhesiveness of hydrogels can attach to tissues and cover wound surface for better closure. At the same time, it can be used as adhesives, haemostatic agents or sealants for reducing bleeding time and blood loss. Ce@LTA-NPs-F127/CS hydrogels as nanoparticles loaded hydrogels are effective targeted therapeutic agents for diabetic wound healing. However, more in-depth research and further optimization studies will be carried out to improve the surface area of Ce@LTA-NPs and graft the targeted functional groups on the surface of the Ce@LTA-NPs to promote the clinical application of our prepared nanoparticles loaded hydrogels.

## Conclusion

A Ce copped zeolite-based nanoparticles characterized by its good porosity, stability and biocompatibility promoted endothelial cell proliferation and migration in vitro, and the obtained antibacterial Ce@LTA-NPs-F127/CS hydrogels improved wound healing in diabetic rats by increasing neovascularization and remodeling microenvironment of the wound site. We further identified that the Ce@LTA-NPs neutralized detrimental inflammatory factors via absorption and eliminated ROS by the Ce-mediated SOD and CAT mimetic catalytic activities. Our results suggested that Ce@LTA-NPs-F127/CS hydrogels with enhanced neutralization of detrimental factors and ROS scavenging effects are an effective therapeutic agent for accelerating diabetic wound repair.

## Supplementary Information

**Additional file 1.** (A) FTIR spectra of Ce@LTA-NPs (B) UV spectra of Ce@LTA-NPs (C) DSC curve of Ce@LTA-Ps.

**Additional file 2.** Quantification of length of diabetic wound at day 7 and 14 (scale bar: 5 mm).

## Data Availability

All data generated or analyzed during this study are included in this published article.

## Abbreviations

SOD: Superoxide dismutase
CAT: Catalase
LTA: Linde type A
CS: Chitosan
Ce@LTA-NPs: Ce doped Linde type A zeolite based nanoparticles
Ce@LTA-NPs-F127/CS hydrogel: Ce doped Linde type A zeolite based nanoparticles loaded hydrogel composed of Pluronic F127 and chitosan
ROS: Reactive oxygen species
HUVECs: Human umbilical vein endothelial cells
