# Supplementary figures and images for: A thermoreversible antibacterial zeolite-based nanoparticles loaded hydrogel promotes diabetic wound healing via detrimental factor neutralization and ROS scavenging
Source: J Nanobiotechnology. 2021 Dec 11;19:414. doi: 10.1186/s12951-021-01151-5 (PMC8665638; doi:10.1186/s12951-021-01151-5)

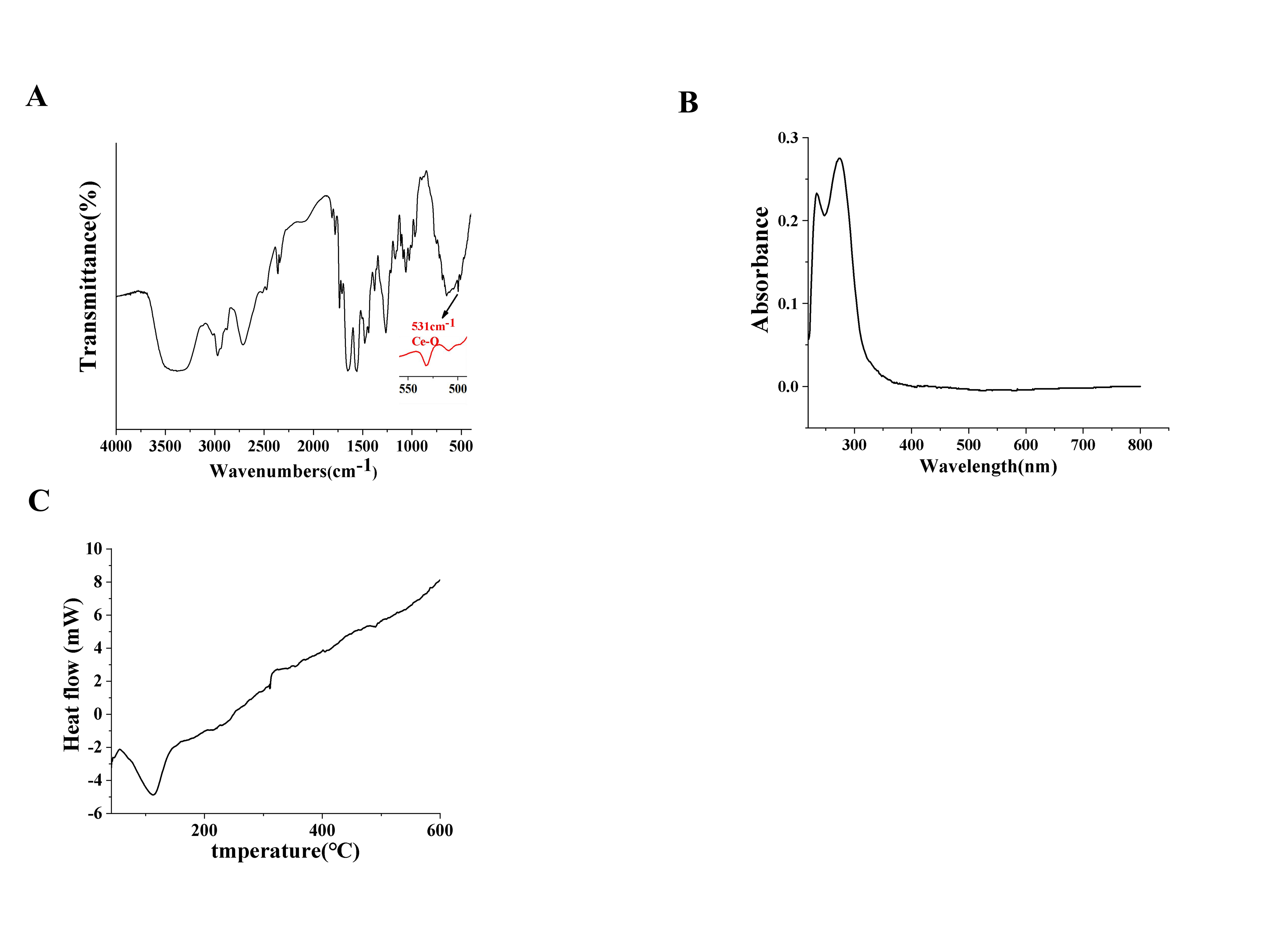

Supplement: Supplementary file 1 — Additional file 1. (A) FTIR spectra of Ce@LTA-NPs (B) UV spectra of Ce@LTA-NPs (C) DSC curve of Ce@LTA-Ps. [file 12951_2021_1151_MOESM1_ESM.png]

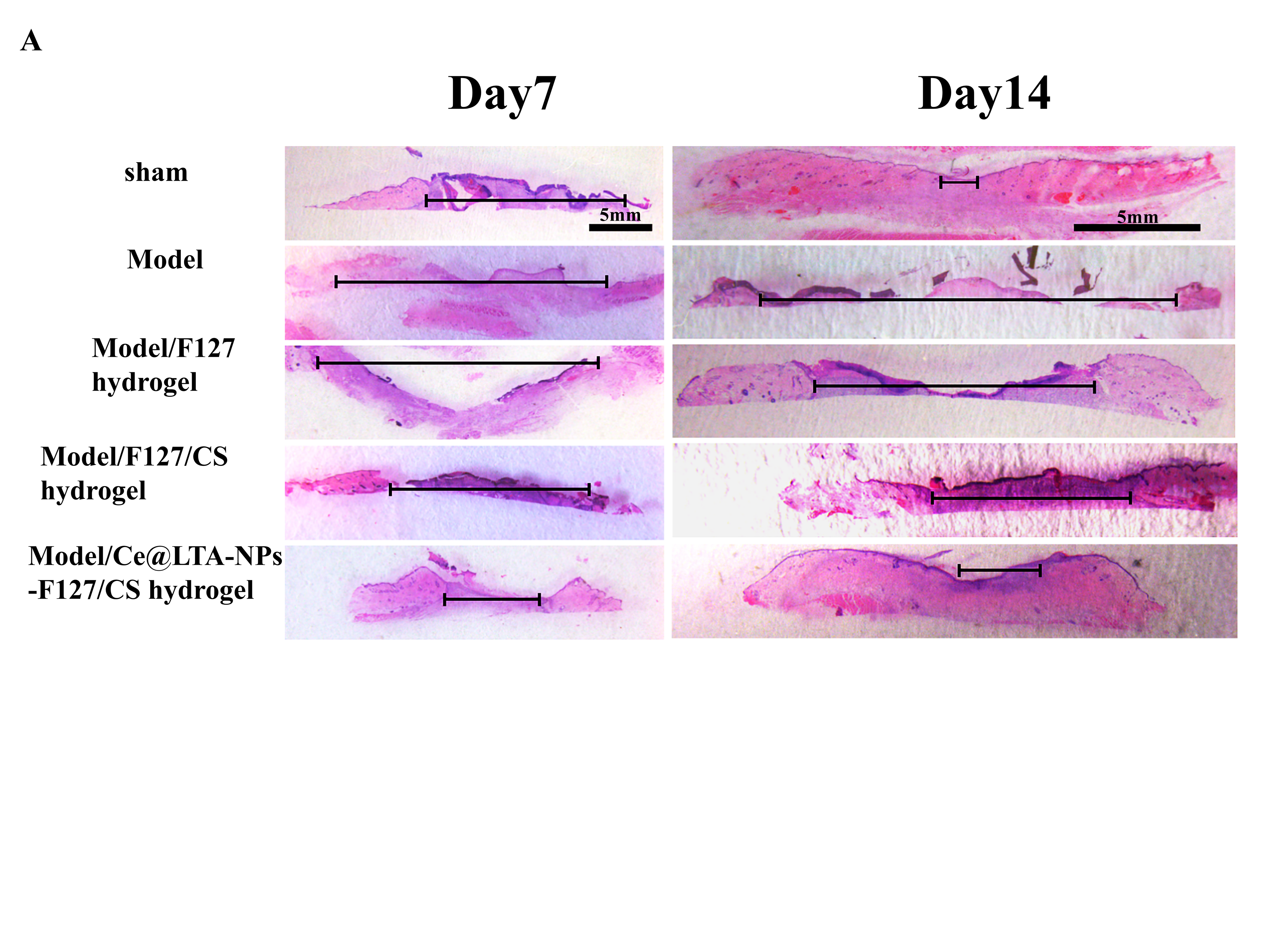

Supplement: Supplementary file 2 — Additional file 2. Quantification of length of diabetic wound at day 7 and 14 (scale bar: 5 mm). [file 12951_2021_1151_MOESM2_ESM.png]
